# Position statement of Italian Society of Obesity (SIO): Gestational Obesity

**DOI:** 10.1007/s40519-024-01688-y

**Published:** 2024-09-27

**Authors:** Luigi Barrea, Stefania Camastra, Silvia Garelli, Valeria Guglielmi, Melania Manco, Fernanda Velluzzi, Rocco Barazzoni, Ludovica Verde, Giovanna Muscogiuri

**Affiliations:** 1 Dipartimento Di Benessere, Nutrizione E Sport, Centro Direzionale, Università Telematica Pegaso, Via Porzio, Isola F2, 80143 Naples, Italy; 2 Unità di Endocrinologia, Diabetologia e Andrologia, Dipartimento di Medicina Clinica e Chirurgia, Centro Italiano per la cura e il Benessere del Paziente con Obesità (C.I.B.O), Università degli Studi di Napoli Federico II, Via Sergio Pansini 5, 80131 Naples, Italy; 3 Department of Clinical and Experimental Medicine, University of Pisa, 56126 Pisa, Italy; 4 Division of Endocrinology and Diabetes Prevention and Care, IRCCS Azienda Ospedaliero-Universitaria di Bologna, Bologna, Italy; 5 Unit of Internal Medicine and Obesity Center, Department of Systems Medicine, Policlinico Tor Vergata, University of Rome Tor Vergata, Rome, Italy; 6 Predictive and Preventive Medicine Research Unit, Bambino Gesù Children’s Hospital IRCCS, Rome, Italy; 7 Obesity Unit, Department of Medical Sciences and Public Health, University Hospital of Cagliari, Cagliari, Italy; 8 Department of Internal Medicine, Trieste University Hospital, Trieste, Italy; 9 Department of Public Health, University of Naples Federico II, Via Sergio Pansini 5, 80131 Naples, Italy; 10 Unità di Endocrinologia, Diabetologia e Andrologia, Dipartimento di Medicina Clinica e Chirurgia, Università degli Studi di Napoli Federico II, Via Sergio Pansini 5, 80131 Naples, Italia; 11 Cattedra Unesco “Educazione alla Salute e Allo Sviluppo Sostenibile”, Università degli Studi di Napoli Federico II, Via Sergio Pansini 5, Naples, Italia

**Keywords:** Pregnancy, Obesity, Gestational obesity, Gestational weight gain, Gestational diabetes mellitus, Pregnancy, Nutrition, Diet

## Abstract

**Purpose:**

Gestational obesity (GO) presents a multifaceted challenge to maternal and fetal health, with an escalating prevalence and far-reaching consequences extending beyond pregnancy. This perspective statement by the Italian Society of Obesity (SIO) provides current insights into the diagnosis, maternal and fetal impacts, and treatment strategies for managing this pressing condition.

**Methods:**

This article provides a comprehensive review of the maternal and fetal effects of GO and provides suggestions on strategies for management. Comprehensive review was carried out using the MEDLINE/PubMed, CINAHL, EMBASE, and Cochrane Library databases.

**Results:**

The diagnosis of GO primarily relies on pre-pregnancy body mass index (BMI), although standardized criteria remain contentious. Anthropometric measures and body composition assessments offer valuable insights into the metabolic implications of GO. Women with GO are predisposed to several health complications, which are attributed to mechanisms such as inflammation and insulin resistance. Offspring of women with GO face heightened risks of perinatal complications and long-term metabolic disorders, indicating intergenerational transmission of obesity-related effects. While nutritional interventions are a cornerstone of management, their efficacy in mitigating complications warrants further investigation. Additionally, while pharmacological interventions have been explored in other contexts, evidence on their safety and efficacy specifically for GO remains lacking, necessitating further investigation.

**Conclusion:**

GO significantly impacts maternal and fetal health, contributing to both immediate and long-term complications. Effective management requires a multifaceted approach, including precise diagnostic criteria, personalized nutritional interventions, and potential pharmacological treatments. These findings underscore the need for individualized care strategies and further research to optimize outcomes for mothers and their offspring are needed. Enhanced understanding and management of GO can help mitigate its intergenerational effects, improving public health outcomes.

*Level of evidence*: Level V narrative review.

## Introduction

Gestational obesity (GO) is a complex condition that poses maternal and fetal health at significant risks [1]. Its prevalence is steadily increasing, with consequences that extend beyond the gestational period, affecting the long-term health of both mother and child.

The adoption of standard cut-offs to define obesity in pregnancy is still debated, with several organizations proposing varying criteria [2]. Anthropometric assessment is critical to detecting GO, but an analysis of body composition could provide additional information on the severity and metabolic implications of this condition [3].

Women with GO are at risk of developing a number of complications, including gestational diabetes (GDM), gestational hypertension, preeclampsia, and difficulties during labor and delivery [1]. In particular, studies suggest an association between GO and an increased risk of short- and long-term complications for the mother, with mechanisms involving chronic inflammation, insulin resistance, and metabolic dysfunction [4]. In addition, infants born to mothers with GO face a significant risk of perinatal complications, including macrosomia, neonatal hypoglycemia, and an increased risk of birth defects [1]. Long-term effects include an increased risk of childhood obesity, type 2 diabetes, and cardiometabolic diseases, suggesting intergenerational transmission of the metabolic consequences of GO [5].

Nutritional interventions are a key component of GO management, with an emphasis on a balanced diet, calorie intake control, and body weight regulation [6]. However, the effectiveness of such interventions in reducing GO-related complications is still subject to evaluation. Finally, pharmacological approaches for obesity, although promising, are not currently available for women with GO, requiring further study to determine their efficacy and safety in this vulnerable population.

Thus, this position statement of the Italian Society of Obesity (SIO) aimed to provide a comprehensive overview of the current knowledge on GO, focusing on diagnosis, maternal and fetal effects, and available therapeutic interventions. Moving from these evidence, the main purpose of this position statement was to inform healthcare professionals, including physicians, midwives, dietitians, and researchers, about the clinical implications and best practices to be adopted in the management of this critical condition.

## Diagnosis of gestational obesity

### Pre-pregnancy obesity

Excess adiposity is commonly measured through anthropometric methods and expressed as body mass index (BMI), obtained from the ratio of body weight in kilograms to height in square meters (kg/m^2^). In the pre-pregnancy period, as in other life course phases, the diagnosis of obesity is based on the calculation of BMI with reference to the internationally used World Health Organization classification [7].

Pre-pregnancy BMI is an important predictor of maternal and neonatal health outcomes. A high BMI at the beginning of the pregnancy can affect the course of gestation and the outcomes of childbirth. Since the resulting GO seems to negatively affect both the health of the mother and the health of the fetus, international guidelines agree that optimal control of obesity should begin before pregnancy [2, 6, 8–11]

In many countries, including Italy, epidemiological data on pre-pregnancy BMI are scarce. In Italy, statistics report a constant increase in weight excess in adult female population (age range: 18–69) and suggest that around 22% of women in the reproductive years (18–44 years of age) are likely to have a BMI above the normal range (18.5–24.9 kg/m2) [10, 12]. Recently, a study conducted in the Friuli Venezia Giulia region of Italy between 2017 and 2018 found that a significant portion of pregnant women faced issues related to overweight and obesity [13]. At the beginning of pregnancy, 17.7% and 8.2% of women were classified as overweight or with obesity, respectively [13].

Pre-pregnancy weight loss has proven to be an effective intervention to improve medical comorbidities [14, 15] and it should be encouraged, especially in view of the fact that not only large weight reductions, as in the case of bariatric surgery [16], but even small pre-pregnancy weight reductions in women with obesity may be associated with improved pregnancy outcomes [8].

### Anthropometric assessment

The most common anthropometric measures used during pre-conception and pregnancy to evaluate maternal body composition and changes throughout pregnancy are BMI (most often pre-pregnancy) and gestational weight gain (GWG) [17]. A meta-analysis about maternal anthropometry and pregnancy outcomes conducted by the WHO confirmed the inherent value of maternal weight, height, arm circumference, and BMI as a predictor of specific infant and maternal outcomes [17].

In particular, GWG has been related not only to fetal growth and newborn weight [18] but also to the risk of intrauterine growth retardation, low birth weight, and prematurity [12]. GWG is defined as the weight gained during pregnancy and up to the pre-delivery visit compared to the weight documented at the first prenatal visit that occurs due to several modifications, including the enlargement of the uterus, fetal development, the formation of amniotic fluid and the placenta, the increase in intra- and extravascular fluids, the breast enlargement, and the fat store [19]. Placenta, fetus, and amniotic fluid generally represents approximately 35% of the total GWG [19].

Currently, recommendations for GWG are based on pre-pregnancy BMI [19] (see Table 1). Specifically, it is  recommended a total GWG range of 7.0–11.5 kg for women with overweight and a total GWG range of 5.0–9.0 kg for women with obesity (BMI > 30 kg/m^2^) regardless of obesity class. Given the limitation of evidence by class of obesity, the general IOM recommendation for women with BMI > 30 kg/m^2^ tries to balance the risk of large- vs. small-for-gestational-age infants and high postpartum weight maintenance vs. preterm delivery [19]. The lack of specific guidance on GWG for classes of obesity is a much-debated topic among specialists in the field, and the literature data provide contrasting results [18, 20, 21]. The literature is unanimous in stating that GWG above the IOM recommendations is associated with an increased risk of adverse maternal and infant outcomes and a long-term effect on maternal health, with an increased risk of mortality for cardiometabolic diseases [18, 20, 21]. On the contrary, the debated question is whether the recommended 5- to 9-kg GWG for women with BMI > 30 kg/m^2^ should require an additional restriction for women with BMI greater than 35 kg/m^2^ and weight maintenance or even weight loss for women with BMI greater than 40 kg/m^2^ [2].

**Table 1:** Recommendations on gestational weight gain according to pre-pregnancy weight class and BMI

Pre-pregnancy weight class and BMI (kg/m^2^)	IOM Recommendation 2009 [19]		Voerman E et al. 2019^a^ [12]	Devlieger R et al. 2020^b^ [22]	Kiel DW et al. 2007^c^ [23]
	Recommended WG range (kg)	Recommended WG in 2° and 3° trimester range (kg/wk)	Suggested WG range (kg)	Suggested WG (kg)	Suggested WG range (kg)
Underweight (< 18.5 kg/m^2^)	12.5–18.0	0.44–0.58 (0.51)	14–16	21	-
Normal weight (18.5–24.9 kg/m^2^)	11.5–16.0	0.35–0.50 (0.42)	10–18	14	-
Overweight (25.0–29.9 kg/m^2^)	7.0–11.5	0.23–0.33 0.28	2–16	8	
Obesity (≥ 30 kg/m^2^)	5.0–9.0	0.17–0.27 (0.22)			
Obesity Class 1 (30.0–34.9 kg/m^2^)	–	–	2–6	0	4.5–11.4
Obesity Class 2 (35.0–39.9 kg/m^2^)	–	–	0–4	Loss-4	0–4.1
Obesity Class 3 (≥ 40 kg/m^2^)	–	–	0–6	Loss-5	Loss 0–4.1

BMI, body mass index; IOM, Institute of Medicine; WG, weight gain

^a^WG based on risk reduction in any adverse outcome

^b^WG associated with the lowest risk of pregnancy complications

^c^WG based on minimal risk of all outcomes

### Body composition assessment

GWG and BMI have been proposed as screening methods for identifying pregnancies with abnormal progression that might be at risk of adverse perinatal outcomes. Nonetheless, they provide very limited information regarding changes in women’s body compositions throughout their pregnancies. Moreover, fetal growth may be influenced by more specific maternal tissue changes (fat or fat-free mass) than by total GWG or BMI [24].

Maternal fat mass (FM) is the most variable component of GWG, and its changes are positively correlated with GWG and inversely related to pre-pregnancy BMI [3]. Based on serial measurements in pregnant women, most of the FM deposited during pregnancy is subcutaneous, with a preferential accumulation over the hips, back, and upper thighs up to about thirty weeks’ gestation. During pregnancy, a large and variable accumulation of water contributes to GWG and determines an increase in the hydration of fat-free mass (FFM) [3]. In the second trimester, GWG is mainly due to FM accumulation, while in the third trimester, it is mainly due to FFM accumulation and fetal growth at the expenses of fat accumulation suggesting that the second trimester is a more opportune window for the assessment of GO because it is characterized by fat accumulation [25].

Several methods could be used for evaluating maternal body composition and to assess changes in FM and FFM before, during, and after pregnancy, and several studies have been performed to compare the link of the measures assessed by different techniques with maternal and child outcomes [26]. Methods used to assess body composition include skinfold thickness (SFT), bioimpedance analysis (BIA), air displacement plethysmography (BOD POD), underwater weighing, and isotope dilution. Simple and cost-effective techniques such as SFT and BIA, a more accurate but expensive method like BOD POD, are all influenced by the hydration of FFM [26].

Equations exist to estimate the percentage of body fat using SFT in order to adjust the data for fluid shifts; however, they are specific to certain gestational stages [27]. Despite these limitations, SFT has shown a high correlation with the percentage of body fat obtained through other techniques (densitometry, DEXA, or dilutional methods) [26]. Thus, this method is useful to describe normal body fat changes throughout gestation, to identify women with unusually small or large changes in body fat during pregnancy, and to estimate initial body fat content [24].

BIA is used to assess the body composition and hydration status. This technique represents a non-invasive, reliable, and fast clinical approach, that is well tolerated and widely accepted by patients. BIA measurements should not be taken when participants are dehydrated, within 4 h of consuming food and drink, or within 12 h of intense exercise [28]. However, the main limit of BIA is that it cannot distinguish between the maternal and fetal tissues [27]. However, despite the advantages of BIA, the abnormal fluid distribution during pregnancy renders different BIA methods either inappropriate or in need of further validation [29]. The whole-body impedance is mainly predicted by the impedance in the limbs [30]; however, during pregnancy, a large amount of water is in the trunk [29]. Thus, a segmental impedance measurement might be advantageous for pregnant women, particularly in late pregnancy [29, 30] (see Table 2).

**Table 2:** Body composition assessment methods in pregnancy

Method	Measures	Strengths	Weaknesses
Skinfold thickness	Estimate of FM Use of skinfold thickness measurements themselves rather than estimates of FM and FFM are often preferred	Simple, inexpensive	Equations to estimate total BF, taking into account the change in hydration of the FFM, only available for certain gestational ages
Bioelectrical impedance analysis	TBW, FM, FFM, ECW, ICW	Simple, inexpensive	Equations to estimate total BF only available for certain gestational ages; unable to disentangle the maternal–fetal unit
Underwater weighing	Body volume (estimate FM and FFM)	Accurate	Method requiring complex equipment. Unable to disentangle the maternal–fetal unit. Results are influenced by changes in hydration of FFM during pregnancy
Air displacement plethysmography (ADP/BOD POD)	Body volume (estimate FM and FFM)	Accurate	Expensive and requiring complex equipment. Unable to disentangle the maternal–fetal unit. Result influenced by changes in hydration of FFM during pregnancy
Isotopic dilution	TBW (estimate FFM)	Accurate	Require frequent urine collection. For research only. It is expensive and requires complex equipment and experienced technicians
Whole-body potassium counting	BCM, FFM	Accurate	It is expensive and requires complex equipment; less accurate during pregnancy due to variation in postassi content in the FFM
Magnetic resonance imaging	FM, FFM, Ectopic FAT, FAT distribution	Accurate	For research only. It is expensive and requires complex equipment and experienced technicians
Dual energy X-ray absorptiometry	FM, FFM, Bone density	Contraindicated during pregnancy due to the radiation exposure	

BF: body fat; TBW total body water; FM fat mass; FFM fat-free mass; ECW extracellular water; ICW intracellular water; BCM body cell mass

However, due to the dynamic state of pregnancy, there is no agreement on the optimal method for quantitatively assessing body fat in pregnant women. The validation of algorithms that regulate the hydration changes observed in the various gestational stages would be necessary.

In addition to body composition, changes in adipose tissue distribution throughout pregnancy could have a differential impact on metabolic health and adverse outcomes [31, 32]. Imaging technologies such as magnetic resonance imaging (MRI) are accurate but expensive. Less expensive is the ultrasound technique that permits the measurement of subcutaneous (SAT), visceral (VAT), and total adipose tissue depth. In this contest, VAT and total adipose tissue at 11–14 weeks of pregnancy have been found to be associated with GDM at 24–28 weeks’ gestation [31]. In addition, simple and inexpensive anthropometric measures such as neck circumference, waist circumference, hip circumference, arm circumference, and waist-to-hip ratio were related to the risk of GDM [32, 33].

## Maternal effects of gestational obesity

### Short-term effects

Obesity during pregnancy represents a risk factor for spontaneous abortion and for complications through the gestational period and delivery [34]. Maternal overnutrition and obesity are associated with multiple modifications that can harm the health of mothers, inducing hypertensive disorders, GDM, and affecting labor and delivery [34].

Based on wide cohort studies, the incidence of any maternal complication increases with BMI and with GWG; in particular, more than 60% of women with grade III obesity develop at least one pregnancy complication, regardless of weight gain [12]. This evidence is related to the definition of optimal ranges of GWG according to pre-conception weight status [19]. Interestingly, a recent systematic review emphasizes the superiority of adiposity markers, in particular waist circumference and waist-to-hip ratio, as strong predictors of adverse maternal health outcomes [33].

GDM represents one of the most frequent complications in mothers with obesity, and it predisposes to large for gestational age, fetal macrosomia, and cesarean delivery [35]. Worldwide, it has been estimated that the prevalence of GDM is about 18.3% and more when considering the female population with obesity, which has been associated with a three-fold risk of GDM [36, 37]. Many epidemiological studies indicated overweight and obesity as main determinants in GDM development, in addition to non-white ethnicity, maternal age, family or personal history of GDM, type 2 diabetes mellitus, subfertility, and infertility [38–40]. Moreover, obesity predisposes to early onset of GDM compared to healthy women; therefore, Italian guidelines for diabetes care included BMI ≥ 30 kg/m^2^ within high-risk conditions for GDM justifying anticipated screening with a 75 g oral glucose tolerance test (OGTT) at the 16–18th week [41]. If negative, the test must be repeated at the 24–28th week [41].

Obesity and GMD are frequently associated with hypertensive disorders. Further, gestational hypertension and preeclampsia are the leading causes of both maternal and fetal mortality worldwide and have been associated with the development of premature cardiovascular diseases and mortality in the offspring [42, 43]. It has been demonstrated that women with abnormal echocardiograms are at an increased risk of hypertensive disorders, in particular severe preeclampsia [44].

When considering delivery, GO and consequent fetal macrosomia have been associated with a longer first stage of labor (usually independent of fetal size), an increased risk of prolonged inductions of labor and induction failure, and shoulder dystocia, resulting in higher rates of cesarean birth (ranging from 18.7 to 43.9% in different cohort studies) [45, 46]. Besides, during puerperium, obesity predisposes not only to genital and wound infections [47, 48], deep venous thrombosis, and pulmonary embolism [49, 50] but also to mental health problems [51].

The pathogenic role of obesity during pregnancy is not completely clarified; however, accumulating evidence has demonstrated its capacity to induce inadequate adaptation of the organism during pregnancy, thus promoting and anticipating the onset of short- and long-term complications.

Pregnancy is associated with GWG, owing to fat accumulation, increased circulating blood volume, and extracellular fluid expansion; moreover, it involves cardiovascular and metabolic modifications to sustain energy delivery for fetus development and growth [52, 53]. Hemodynamic adaptations include an increase in heart rate, stroke volume, and cardiac output, cardiac hypertrophy, increase in plasma volume, decrease in arterial and peripheral vascular resistance, and decrease in blood pressure levels [54]. Metabolic change differentiates during pregnancy: the first half of pregnancy is characterized by higher insulin sensitivity, an anabolic state, and increased lipid storage; during the second half, insulin sensitivity reduces and cortisol, prolactin, human placental lactogen levels rise, leading to a catabolic state with an increased blood concentration of glucose, amino acids, and lipids available for the fetus. The development of insulin resistance is usually balanced by an increased rate of insulin secretion due to β-cell hyperplasia, which contributes to the maintenance of normal maternal glucose levels [54]. In this scenario, maternal central adiposity, insulin resistance, and inflammation appear to play central roles in the pathogenesis of pregnancy and delivery complications.

In particular, VAT accumulation is associated with worsening of insulin resistance and derangements of insulin signaling, disturbance of lipid metabolism, modulation of the inflammatory and immune responses, pro-thrombotic state, and induction and activation of the angiotensin I–II system [55, 56]. VAT acts like an endocrine organ, producing a great amount of adipokines with pro-inflammatory activity (leptin, visfatin, and resistin) and tumor necrosis factor-alpha (TNF-α), while anti-inflammatory adiponectin is reduced [57]. This is responsible for the increase of specific cytokines, such as interleukin (IL)-1β, IL-2, and IL-6, promoting the expression of maternal antiangiogenic factors and supporting the development of insulin resistance and systemic inflammation [58]. Furthermore, hyperleptinemia has been directly involved in placental development and function; it reduces cytotrophoblast proliferation and placental growth factor (P1GF), contributing to placental ischemia and early preeclampsia [59].

In mothers with visceral obesity, insulin resistance is compounded compared to healthy women and β-cell secretory function is insufficient to tackle the enhanced hormonal demand [60]. Moreover, low dietary intake of fiber and a sedentary lifestyle, as well as the pre-existence of obesity comorbidities, such as polycystic ovary syndrome, can contribute to worsening glucose metabolism during pregnancy. The overall results are progressively growing glucose and advanced glycation end products (AGEs) levels in mothers, while the development of macrosomia, fat deposition, insulin resistance, and neonatal hypoglycemia in babies [60]. Recent studies conducted with lipidomic techniques in pregnant women confirm that insulin resistance is associated with altered lipid metabolism toward lipogenesis, resulting in increased free fatty acids and cholesterol flow [61], a reduction of sphingomyelin, and polyunsaturated fatty acid-rich lipids [62]. Free fatty acids contribute to induce endothelial dysfunction and pro-thrombotic state (reduction of nitric oxide and prostacyclin, increase of reactive oxygen species, thromboxane A2, and endothelin 1), which overlap with vessel modification induced by hyperinsulinemia (vessel wall hypertrophy, narrowing of the vessel lumen, and increased peripheral resistance), collectively leading to hypertension [62]. High low-density lipoprotein (LDL) cholesterol and low high-density lipoprotein (HDL) cholesterol levels have been associated with the deposition of atherosclerotic plaques in the placental vasculature and coronary system, contributing to the increased cardiovascular risk in mothers [63]. Finally, hyperinsulinemia influences innate immune system function involved in the pathogenesis of preeclampsia. The complement system is activated by lipid (primarily non-esterified fatty acids) accumulation in the placenta [64]. Further, hyperinsulinemia increases the expression of M1 macrophages and modulates the mammalian target of rapamycin complex 1 (mTORC1) pathway, thus inducing an imbalance between pro-inflammatory (increased) and anti-inflammatory (reduced) T-lymphocyte populations [65], overall contributing to heightening both systemic and placental inflammatory states; furthermore, it acts locally at the maternal–fetal interface, reducing the number of decidual natural killer cells (dNKs) and vascular endothelial growth factor (VEGF) production while increasing TNF-α [58].

In conclusion, the data highlight the negative impact of visceral obesity in the progression of pregnancy and, thereafter, on women’s and babies’ health. In particular, the presence of visceral adiposity seems to be pivotal in inducing systemic and local inflammation, immune system derangement, oxidative stress, insulin resistance, antiangiogenic effects, and endothelial damage, all of which are associated with clinical maternal and fetal manifestations. Based on these observations, it is crucial to put in place strategies for prevention, diagnosis, monitoring, and treatment of obesity complications, starting from the pre-conception period, as endorsed by the International Federation of Gynecology and Obstetrics guidelines [11, 66].

### Long-term effects

#### Breastfeeding

Obesity and overweight are associated with low rates and shorter durations of breastfeeding [67, 68] compared with women of normal weight, and this can further contribute to later adverse health outcomes that are associated with obesity, including breast and endometrial cancer [69, 70] in the menopausal period and type 2 diabetes [71, 72] as well as postpartum weight retention [73]. Delayed onset of lactation, due to high rates of cesarean section [74] and elevated progesterone levels (which prevent lactogenesis), is implicated [75], but also physical discomfort due to heavy breasts and postnatal depression may be potential contributory factors [76]. Breastfeeding should be encouraged because it may exert protective effects against later obesity-related adverse outcomes for the mother and offspring.

#### Postpartum weight retention

Pregnancy has been recognized as an independent risk factor for both persistent and new-onset obesity in women [77]. Most women gain rather than lose weight between pregnancies [78–80] and GWG is the main predictor of postpartum weight retention [81–83] as well as an important contributor to the increasing prevalence of obesity in women of reproductive age [84]. Indeed, women with excessive GWG have a 2–4.5-fold higher risk of being affected by overweight or obesity at 21 years postpartum, respectively [82]. Independent of GWG, pre-conceptional BMI seems to impact postpartum weight retention [85, 86] with a 0.10 kg increase in weight retention per unit of baseline BMI at the beginning of a second pregnancy [87]. In addition, there is some evidence suggesting regional differences in fat accumulation during postpartum, with a preferentially central rather than peripheral distribution in women with obesity compared to their normal weight counterparts [88, 89], with important implications for the development of later obesity complications. Finally, it should acknowledge that regardless of GWG, trajectories of weight gain over life span reveal greater gains among women with children compared to women with no births [90, 91].

#### Long-term morbidity

Whether postpartum weight retention negatively influences the risk of long-term complications of GO and the maternal and fetal outcomes in the next pregnancy, the complications of obesity during pregnancy represent a further threat to the long-term health of women [92, 93]. Indeed, women with GDM [92], raised HbA1c, or fasting glucose concentrations [94] are at increased risk not only for recurrent GDM [95] but also for developing type 2 diabetes after delivery, with a sevenfold higher incidence of type 2 diabetes in the first decade compared with non-complicated pregnancies [96], as well as BMI ≥ 25 kg/m^2^, family history of diabetes, non-white ethnicity, multiparity, and older maternal age [94]. Maternal derangements of plasma glucose are an increasingly recognized risk factor even for future cardiovascular disease, with a 13% higher risk of cardiovascular disease for every 1 mmol/L increment in the glucose challenge test even in the non-diabetic range [97]. Similarly, after preeclampsia, a fourfold higher risk of hypertension and a doubled risk of ischemic heart disease and cerebrovascular disease have been reported [98]. A pathogenetic interplay between pre-pregnancy heightened cardiovascular risk and cardiometabolic effects of hypertensive disorders of pregnancy such as endothelial dysfunction, inflammation, dyslipidemia, and angiogenic changes leading to accelerated atherogenesis that may sustain in the postpartum period has been hypothesized [76].

The postpartum increase in central adiposity predisposes women with obesity to hypertension and alterations in serum lipids, emerging as early as 1 year after delivery and persisting for more than 10 years [99, 100].

Women with GO developing pregnancy complications should undergo postpartum follow‐up for prevention, diagnosis, and timely management of chronic diseases.

## Fetal effects of gestational obesity

### Short-term effects

#### Perinatal complications

An increased BMI in pregnancy is associated with an increased risk of maternal morbidity and mortality in the perinatal period, with a dose–response relationship [101]. A retrospective population-based study on a large cohort of women from Washington State found a progressive higher risk of severe maternal complications, from class I to class III pre-pregnancy obesity compared with a normal BMI. Overall, women with an elevated BMI had a significantly higher risk of antepartum or postpartum hemorrhage, sepsis, thromboembolism, cardiovascular or respiratory morbidity, acute renal failure, eclampsia, complications of obstetric intervention, and maternal death. Additionally, women with obesity also had a higher rate of previous infant death, preterm birth, labor induction, delivery complications, and cesarean delivery [102]. As for delivery complications, in addition to the increased risk of hemorrhage and sepsis post vaginal birth, GO also predisposes to prolonged labor, an increased requirement and dosage of oxytocin, and higher rates of anesthetic complications, including both epidural and general anesthesia. These complications may negatively impact the newborn, inhibiting the initiation of breastfeeding, a known protective factor for a child’s health [101]. Maternal perinatal death in women with obesity is mainly due to cardiovascular complications [103]. Moreover, obesity has been described as one of the most frequent comorbidities for mortality related to COVID-19 infection, in pregnant women [104]. Furthermore, women with overweight or obesity have a significantly higher risk of developing hypertension and GDM [105], as well as sleep-disordered breathing [106], compared to women with normal weight, and these pathological conditions are associated with adverse impacts on delivery and newborn health. Finally, it has been reported that there is an association between GO and perinatal depression, a condition that includes depressive symptoms both during pregnancy and/or in the postpartum period, which can have a negative influence on newborn care following birth. Although the mechanisms supporting this association are not clear, it has been hypothesized that hypovitaminosis D, or body image disturbance, may play a role [107].

#### Fetal complications

GO during pregnancy has a negative impact on the fetus and represents an independent risk factor for various fetal complications during pregnancy, delivery, and throughout the offspring’s life [101]. GO at conception and the related metabolically unfavorable ovarian follicular environment [108] could negatively influence early embryonic development through metabolic, cellular, and epigenetic mechanisms, since the preimplantation stage, with negative consequences for offspring’s health over the lifetime [109]. The periconceptional environment could also affect babies born as a result of assisted reproductive treatments. In a retrospective observational study investigating supernumerary embryos from women with a BMI > 25 kg/m^2^ undergoing fertility treatment, morphologic and metabolic alterations indicative of a poorer potential in the resulting blastocysts have been described [110]. As regards fetus development, the placenta plays a central role in the exchange of materials between fetus and mother. In the case of GO, an altered adipokine pattern and impaired angiogenesis could compromise placental function, leading to a negative impact on nutrient transport capacity and placental blood flow [111]. This, in turn, can adversely affect fetal growth and health throughout life [112]. On this concern, several pre-clinical and clinical studies support the hypothesis that an inadequate in-utero environment represents a favorable factor in the development of various complications in the offspring [113]. Concerning fetal early complications, perinatal death, congenital fetal abnormalities, and fetal macrosomia are more likely to occur among mothers with obesity compared to those with a normal weight [76]. A systematic review and meta-analysis reported that even a slight increase in maternal BMI was associated with an elevated risk of stillbirth, fetal, perinatal, and neonatal death [114]. Moreover, it has been reported that overweight and obesity are the major modifiable risk factors for stillbirth in high-income countries [115]. A recent population-based study, carried out in a large sample of Swedish women, found a double risk of stillbirth among women with overweight and a fourfold risk among those with obesity, compared with women of normal weight [116], in line with the results of a previous large population-based cohort study [117]. In addition, pregnancy complications associated with GO, such as hypertension, preeclampsia, fetal anomalies, placental diseases, or umbilical cord abnormalities, are predisposing factors to an increased risk for stillbirth as well as for having small-for-gestational-age (SGA) babies [117]. SGA birthweight is a condition strongly associated with neonatal morbidity and mortality, but its relationship with GO is unclear, also depending on the SGA classification method used. In this regard, in a large cohort study, SGA, classified using intrauterine references, was associated with a five-fold increased risk of stillbirth among women with obesity, after excluding those with diabetes or hypertension. However, in the same cohort, women with overweight or obesity had a lower risk of SGA than those with a normal weight [118]. On the other hand, a cohort study in a Chinese population, reported a higher SGA risk among women with pre-pregnancy obesity compared to those with normal weight, hypothesizing a possible role of placental inflammation in obesity [119]. Although the pathogenetic mechanisms are still unclear, it has been suggested that the higher risk of stillbirth among women with obesity could be related to metabolic alterations, inflammatory status, or placental dysfunction associated with obesity [120]. A case–control study comparing the placental characteristics in cases of stillbirth and live-born to women with obesity found an association between stillbirth and umbilical cord alterations [121]. GO, particularly severe obesity, is also associated with a higher risk of spontaneous abortion or recurrent miscarriage [122], cesarean section and subsequent risk of infections [123], preterm delivery, both spontaneous and induced [124], transfer to neonatal intensive care units [125], and a low Apgar score at five minutes after birth [116].

With regard to congenital malformations, a meta-analysis reported an increased risk of various structural anomalies, mainly neurological, cardiac, and limb anomalies, among women with obesity compared to women with a recommended BMI [126]. More recently, a large population-based cohort study found that the risk of major congenital anomalies progressively increases with maternal BMI, rising from overweight to class III obesity [127]. Among neurological malformations, fetal spina bifida is the most common, affecting approximately 1 in 3000 births [128]. The prevalence of this congenital anomaly has been reported to be ten times higher among the offspring of women with overweight or obesity compared to those of women with normal weight [129]. Moreover, obesity presents a substantial clinical concern given that the Management of Myelomeningocele Study eligibility criteria preclude prenatal surgery, considered the optimal treatment for fetal spina bifida, for women with a BMI > 35 kg/m^2^ due to safety issues [130]. However, a recent preliminary single-center study that included women with a BMI > 35 kg/m^2^ in the fetal spina bifida surgery cohort did not find any relevant difference in clinical outcomes compared to women with a lower BMI [131]. Other neurodevelopmental outcomes, such as attention deficit-hyperactivity disorder, autism spectrum disorder, developmental delay, and emotional and behavioral problems, are observed more frequently among infants born to mothers with GO [132]. The inflammatory status, redox imbalance, high levels of oxidative stress, nutritional deficiencies, changes in the maternal gut microbiota, and epigenetic alterations associated with GO could potentially represent causal factors for the abnormalities observed in neural development among newborn of women with obesity [133]. Although there is still little evidence in the human population, pre-clinical studies have shown that chronic inflammation, metabolic and hormonal changes, including elevated insulin and leptin levels, and alterations in the serotonin and dopamine systems associated with obesity can impact brain development and subsequent functioning, leading to neurological and psychological disorders [132, 134]. Congenital heart disease (CHD), characterized by defects in cardiac development within the first six weeks of pregnancy, is the most common congenital abnormality, with an increasing global birth prevalence and a strong clinical and social impact [135]. Among the various factors contributing to CHD susceptibility, a meta-analysis, incorporating 23 studies that assessed the relationship between CHD risk and maternal BMI, found a significant association between GO and elevated CHD risk in offspring [134]. Possible pathogenetic mechanisms include impaired folate and homocysteine levels, as well as metabolic, hormonal, or inflammatory alterations that may adversely affect the intrauterine environment [134]. Moreover, a recent register cohort study conducted in Finland highlighted that gestational overweight or obesity was specifically associated with an increased risk for complex cardiac defects and outflow tract obstruction defects, suggesting the existence of specific teratogenic mechanisms, different from those linked with GDM, for these obesity-related CHD subtypes [136]. Aside from glycemic dysregulation, other possible mechanisms that may contribute to an increased CHD risk in the offspring of women with GO include an abnormal maternal lipid profile, increased oxidative stress, endothelial dysfunction, and endocardial dysfunction [136]. Other studies have observed a positive association between GO and congenital abnormalities of the kidney and urinary tract in offspring [29, 137]. This group of malformations exposes affected children to a high risk of morbidity and mortality [29, 137]. Additionally, GO has been linked to genital malformations, specifically hypospadias and cryptorchidism, in male offspring, although the underlying mechanisms remain unknown [138]. Fetal macrosomia is defined as a birth weight above the 90th percentile for gestational age, or > 4000 g, and has a prevalence ranging from 0.9 to 12% of all pregnancies. Macrosomia increases the risk of various short-term complications, including preterm birth, postpartum hemorrhage, maternal birth canal trauma, cesarean delivery, shoulder dystocia, fetal asphyxia, and neonatal hypoglycemia. Additionally, it increases the risk for long-term complications such as obesity, type 2 diabetes, metabolic syndrome, and in general, non-communicable diseases [139]. Among the predisposing factors, GO, encompassing both pre-pregnancy obesity and excessive GWG shows a strong association with fetal macrosomia [140]. Maternal insulin resistance and subsequent hyperglycemia associated with obesity are major determinants of fetal macrosomia, leading to increased placental glucose transport and fetal insulin hypersecretion. Insulin, through its anabolic effects, promotes excess fetal growth [141]. Moreover, GO is associated with an increased placental weight compared to women with normal weight [142] and placental weight is associated with birth weight [143]. Finally, it has been reported that there is a higher incidence of viral and bacterial infections among newborns of women with obesity, suggesting a detrimental effect of GO on the fetal immune system [144].

### Long-term effects

Beyond their immediate implications for maternal and offspring health in the short term, GO and inadequate GWG during pregnancy pose significant risks for offspring health in the long term. Extensive experimental literature in animal models, especially non-human primates [145], and studies in humans have shown that offspring born to mothers with obesity compared to offspring of mothers with a healthy weight are more likely to develop obesity, insulin resistance, cardiovascular diseases, type 2 diabetes [146], and also metabolically associated steatotic liver disease (MASLD) later in life [147]. Additionally, GO has been linked to an increased risk of neurodevelopmental, disorders including autism spectrum disorders [148], poorer cognitive performance [149], and allergic disease in offspring [150].

The effects of GO and GWG on offspring health are believed to be influenced by multiple mechanisms that include mainly shifts in epigenetics such as changes in DNA methylation, histone modifications, and microRNA expression [146, 151]. Indeed, exposure to various maternal factors during pregnancy has been demonstrated to induce permanent alterations in fetal metabolic control processes, serotonin and dopamine pathways, lipid peroxidation, and corticosteroid receptor expression [152] through epigenetic modulation. These alterations encompass, but are not limited to, the hypothalamic response to leptin and subsequent regulation of appetite, as well as pancreatic beta cell physiology [153]. The underlying mechanisms are likely multifaceted and may involve maternal metabolic changes such as variations in glucose and fatty acids [154], modifications in maternal hypothalamic–pituitary–adrenal axis activity [155], inflammation, and alterations in hormonal milieu and placental function. Additionally, other factors contributing to persistent metabolic changes over time include modifications in the offspring gut microbiome, resulting in reduced microbial diversity [156] and the production of short-chain fatty acid metabolites, mirroring the mother’s gut microbiota features [157]. The aberrant gut microbiome, known to be associated with GO, provides a key mechanism to explain not only the immune and metabolic consequences [156] on the developing fetus but also the association between GO and the risk of neurodevelopmental disorders in the offspring [151].

Both greater maternal pre-pregnancy BMI, spanning the entire spectrum, and GWG correlate with increased childhood adiposity and unfavorable body fat distribution [53, 158].

A study of 2432 Australians found that greater maternal GWG was associated with higher offspring BMI at age 21, independent of maternal pre-pregnancy BMI [159]. Similarly, a study in Israel involving 1400 mother–offspring pairs showed that higher maternal pre-pregnancy BMI correlated with increased offspring BMI at age 30, with associations of maternal BMI with cardiovascular risk explained by adult BMI [160]. The Helsinki Birth Cohort Study (HBCS) also indicated a positive association between maternal BMI and offspring BMI at age 60, with higher maternal BMI linked to less favorable body fat distribution in female offspring at around age 62 [161].

Research on registries in Finland and birth records in the UK underscores the role of GO during pregnancy in offspring cardiovascular health [162, 163]. Higher maternal BMI has been linked to increased risks of premature mortality and cardiovascular hospitalizations in adult offspring, independent of socioeconomic status [163]. The HBCS further confirms these findings, showing elevated rates of cardiovascular diseases, coronary heart disease, type 2 diabetes, and stroke among offspring of mothers living with obesity [162].

Studies have sought to determine crucial periods of GWG and their impact on childhood outcomes [146, 164, 165]. Among 5,000 UK mother–offspring pairs, GWG in the first 14 weeks was linked to increased offspring adiposity at age 9 [164]. Similarly, a study with 6,000 Dutch mother–offspring pairs found that early-pregnancy weight gain was associated with an adverse cardiometabolic profile in childhood [165], independent of pre-pregnancy weight gain or later pregnancy weight gain. Godfrey et al. speculate that weight gain in early pregnancy, when maternal fat accumulation is a significant component of total weight gain, may be a critical period for childhood cardiovascular risk [146].

As to MASLD, maternal GWG but not pre-pregnancy BMI has been associated with a risk of hepatic steatosis in carriers of 148 M patatin-like phospholipase domain-containing 3 polymorphisms [166].

GO is associated with a rise in childhood asthma and allergic diseases [167, 168]. A meta-analysis involving 108,321 mother–child pairs found that gestational overweight or obesity during pregnancy increased the risks of childhood asthma or wheezing ever and current asthma or wheezing, independent of offspring BMI [167]. Higher maternal GWG was also linked to increased odds of current asthma or wheezing in offspring. However, GO mainly impacts asthma and wheezing, not eczema, sensitization, or hay fever, suggesting tissue-specific effects [168].

While both maternal pre-pregnancy obesity and excessive GWG appear to correlate with elevated blood pressure, unfavorable lipid profiles, and insulin resistance in childhood, there is some indication that these connections are largely influenced by childhood BMI [165, 169]. In fact, despite the potential for an adverse prenatal environment to heighten the risk of non-communicable diseases from childhood onward, its effects can still be mitigated and reversed through intervention, particularly during childhood and adolescence. These life stages present crucial opportunities for modifying and potentially reversing the risk of adult-onset diseases.

**Fig. 1:**
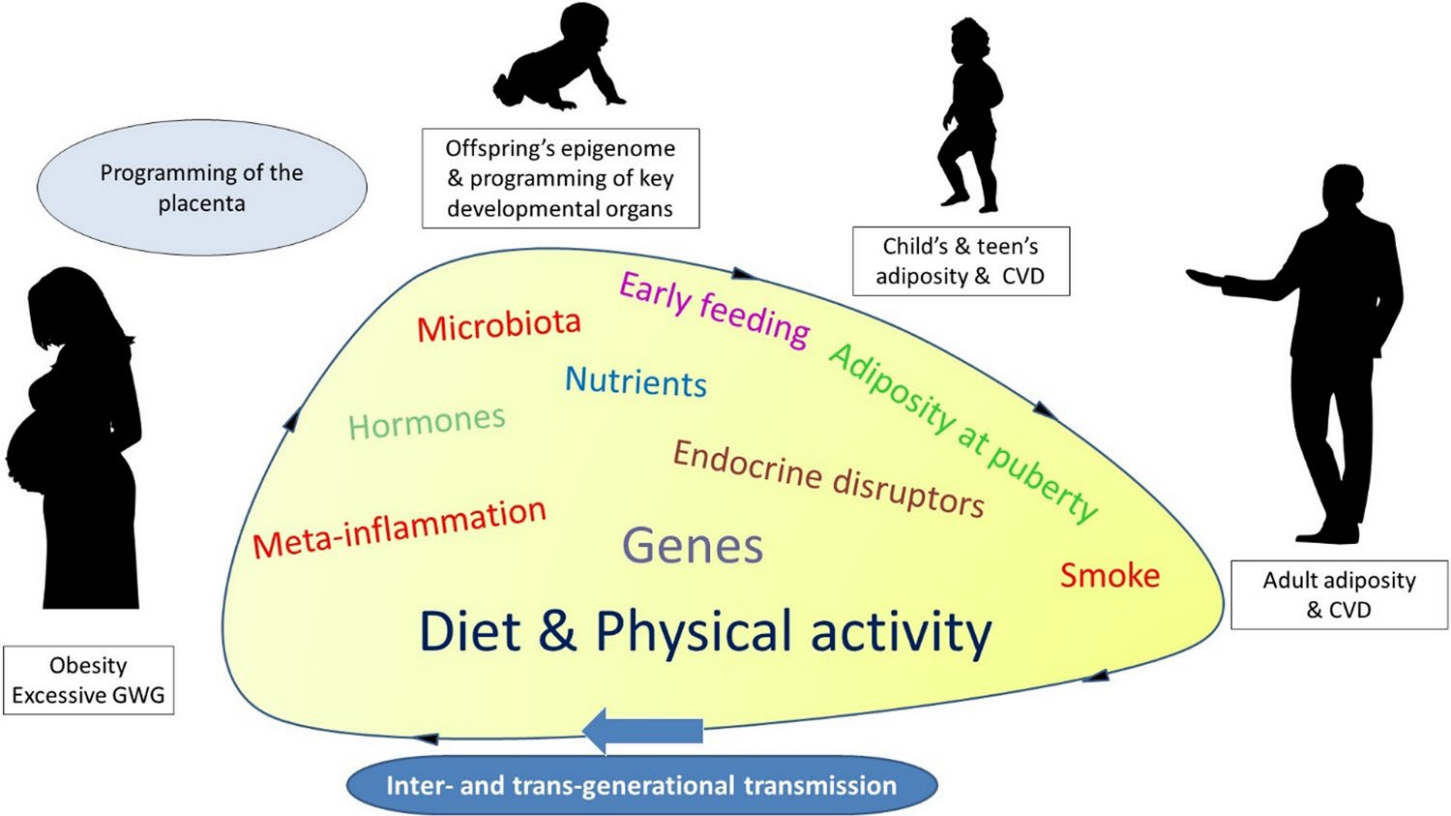
Long-term effects of GO on offspring health

In human studies, a significant hurdle is disentangling intrauterine effects from lingering confounding factors, such as shared genetic variants between mother and offspring, poor dietary choices, sedentary behaviors, and metabolic irregularities, all of which can directly influence fetal development (Fig. 1). Maternal hyperglycemia and excessive GWG are notably impactful risk factors for unfavorable pregnancy outcomes and the health of offspring. Moreover, socioeconomic disparities, including limited healthcare access and insufficient prenatal care, can amplify the impact of GO on offspring health. Postnatal influences on diet/lifestyle behaviors and microbiome-related mechanisms represent additional confounders. Methodological variations, sampling biases, and discrepancies in defining obesity and its outcomes further compound the challenge, often leading to conflicting findings.

## Nutritional intervention for gestational obesity

Nutritional treatment for women with GO is of paramount importance in managing GWG and reducing associated risks. Meta-analyses have found that diet and lifestyle interventions during pregnancy have only a limited effect on reducing GWG [170], but methodological differences between studies in design, dietary regimen, intensity, and measures of compliance make comparison of results difficult. This lack of consensus limits the ability to develop clinical guidelines and apply the evidence in clinical practice.

One of the main weight intervention strategies involves calorie control. Caloric goals typically range from 1200 to 1800 kcal/day for pregnant women with obesity and are adjusted based on factors such as body weight, gestational age, and level of physical activity [171]. For instance, Renualt et al. prescribed a calorie range of 1200–1675 kcal/day for women with GO [172], while Wolff et al. modified calorie goals based on basal metabolic rate and physical activity level [173]. Both interventions demonstrated significant reductions in GWG without adverse effects on maternal or fetal outcomes [172, 173].

Studies have shown that implementing personalized diets tailored to the needs of pregnant women can effectively limit GWG without compromising the health of the mother or fetus [170]. Using maternal and neonatal data on 275,708 full term and singleton cases, Beyerlein et al. concluded that women with GO were safe to have weight loss during pregnancy; their conclusions were based on the association of GWG and a ≤ 20% predicted risk of having small-for-gestational-age and large-for-gestational-age infants [174]. Additionally, the LARN (Reference Intake Levels of Nutrients and Energy for the Italian population) recommend an increase of 350 and 460 kcal per day during the second and third trimesters for pregnant women [175]. Jebeile et al. found that the energy intake of pregnant women remained the same in early and late pregnancy, and therefore, they recommended that additional energy intake be avoided to prevent excessive GWG [174]. By closely monitoring caloric intake, it is possible to prevent excessive GWG and reduce the likelihood of related health complications. However, it is important to ensure that the diet provides adequate nutrients to support the growth and development of the fetus.

In addition to calorie control, optimizing macronutrient composition is essential to managing GO. Studies have examined the effects of different macronutrient ratios on GWG and maternal health outcomes [176, 177]. Macronutrient goals typically include a balance of protein, fats, and carbohydrates, with specific percentages tailored to individual needs. For example, Petrella et al. set macronutrient goals of 20% protein, 25% fat (6% saturated fat), and 55% carbohydrate for women with GO [176]. Similarly, Thornton et al. aimed for a macronutrient distribution of 30% protein, 30% fat, and 40% carbohydrate [177]. These interventions demonstrated significant reductions in GWG among participants who adhered to the prescribed macronutrient goals [176, 177].

Furthermore, promoting healthy eating patterns and behaviors is crucial to managing GO. Recently, high‐quality large‐scale randomized controlled trials have reported that lifestyle interventions during pregnancy that include diet and exercise advice and behavior change support can reduce excessive GWG and the frequency of large‐for‐gestational‐age babies (LIMIT, UPBEAT, PEARS trials) [178–180]. Lifestyle interventions during pregnancy that include diet and physical activity have also been shown to reduce the risk of pregnancy‐induced hypertension, cesarean delivery, and respiratory distress in neonates [181].

In addition to general nutritional strategies, some specific approaches have emerged that can be used in the nutritional treatment of pregnant women with obesity. For example, a study investigated the effects of a high-protein low-glycemic index (HPLGI) diet on GWG, birth weight, and the risk of gestational complications in women with GO [182]. A total of 279 women with GO were randomly assigned to either a HPLGI or a moderate-protein, moderate-glycemic index (MPMGI) diet. The results showed that women in the HPLGI group had a significantly lower GWG compared to those in the MPMGI group, without significant differences in birth weight. The HPLGI group also experienced a lower incidence of pregnancy complications, including a lower incidence of cesarean delivery [182]. Then, a secondary analysis of the same study mentioned above, aimed to evaluate the association between increased animal protein intake and blood pressure changes in pregnant women with overweight or obesity, indicated that increased animal protein intake, along with a low-glycemic index, was not associated with changes in blood pressure [183].

A study investigated the effects of whole blueberry and soluble fiber supplementation on cardiometabolic profiles in women with GO at high risk of developing GDM [184]. The study included 34 women who were randomly assigned to either the intervention group or the control group. The intervention group received daily supplementation of whole blueberries and soluble fiber in addition to standard prenatal care. The results showed that the dietary intervention group had significantly lower GWG compared to the control group. Additionally, markers of inflammation and blood glucose levels were also lower in the intervention group. However, there were no significant differences between the two groups in terms of conventional lipids or infant birth weight [184].

Finally, in GO, managing glycemic control is critical to preventing both hypoglycemia and hyperglycemia [185]. Nutraceuticals like inositol and probiotics have shown promise in this context [185]. Myo-inositol (MI) and D-chiro-inositol (DCI) have insulin-mimetic properties that may reduce GDM risk, especially when taken early in pregnancy [186]. In women with GO, MI (2000 mg twice daily) has shown potential in stabilizing postprandial glucose and reducing glycemic variability, though more extensive research is needed before widespread recommendations can be made [185]. Probiotics may improve insulin sensitivity by modulating gut microbiota, which is particularly relevant in GO. However, evidence is mixed regarding their impact on fasting glucose and GDM prevention, with varying outcomes depending on the strains, dosage, and timing used [185]. While promising, consistent guidelines for probiotic use in this population remain undeveloped.

In conclusion, setting both calorie and macronutrient goals may facilitate healthy eating behaviors and, consequently, better gestational weight management. Specific approaches, such as high-protein, low-glycemic index diets or supplementation with whole blueberries, soluble fiber, and MI, can be used to achieve positive outcomes in gestational weight control and improve maternal and fetal health outcomes. However, although the collective evidence from previous research indicates that healthy eating strategies can help pregnant women with obesity limit excessive GWG, the details of the most appropriate nutritional intervention for these types of women have yet to be studied.

## Pharmacological intervention for gestational obesity

### Anti-obesity medications

All the currently approved anti-obesity medications are not recommended during pregnancy or breastfeeding. Indeed, the lack of adequate data on reproductive outcomes does not allow for definitive statements about their safety in human pregnancy.

Animal studies have suggested teratogenic effects for naltrexone/bupropion, liraglutide, semaglutide, and tirzepatide, except for orlistat, that has shown no embryotoxicity or teratogenicity in rats and rabbits even at supratherapeutic doses [187].

In studies on pregnancy outcomes after inadvertent exposure to anti-obesity medications during unintended pregnancies, the use of orlistat for weight reduction seems not to increase birth defects [188], whereas a positive association between early-pregnancy bupropion use and left outflow tract heart defects [189] and fetal cardiac arrhythmia [190] has been reported. No clinical data on liraglutide and semaglutide exposure in human pregnancies or breastfed infants are available. Due to its long half-life, semaglutide should be discontinued at least 2 months before a planned pregnancy [191] while there is no official recommendation for when to stop liraglutide before pregnancy [192].

### Off-label obesity pharmacotherapy

Metformin has not been officially approved as an anti-obesity medications because its effect on body weight remains inconsistent, although it is often prescribed to improve insulin resistance in non-diabetic subjects with obesity [193, 194]. In contrast to anti-obesity medications, there are relatively few studies on the use of metformin during human pregnancy and breastfeeding, with different results regarding its safety. Metformin readily crosses the placenta, and its use during the first trimester was associated with an increased risk of many birth defects [195], although other studies have not confirmed these associations. Metformin has been reported to reduce GWG and promote weight loss after delivery [193], but it does not appear to reduce the incidence of GDM or improve other direct markers of maternal outcomes [193, 195]. With regard to offspring outcomes, in a recent meta-analysis, metformin exposure resulted in smaller neonates with an acceleration of postnatal growth and a higher BMI in childhood, so guidelines do not recommend metformin as the first-line treatment for GDM [196] and suggest caution, especially in pregnant women with hypertensive disorders or at risk for intrauterine growth restriction [197, 198]. Metformin is detectable in breastmilk, but prospective studies have shown no adverse effects on breastfed infants, although prudence is recommended in mothers with premature infants or with renal impairment [199].

## Conclusion

GO is a threat to both the mother's and fetus’ health and could predispose to the onset of short-term and long-term complications. In order to reduce the risk of developing GO, it is advisable to maintain a healthy diet, exercise, reach a normal weight, and keep GWG within the allowed ranges during pregnancy. Table 3 provides a comprehensive overview of recommendations at each step of pregnancy to manage GO effectively.

**Table 3:** Recommendations for pregnancy management in women with GO

Stage of Pregnancy	Recommendations
Before pregnancy	Risk factors definition: Measure BMI, BP, FBG, and lipids; collect medical history; consider age and ethnicity Counseling: Inform about immediate and long-term risks of obesity; encourage weight reduction (5–10% over 6 months) Treatment: Consider lifestyle intervention, anti-obesity medications, bariatric surgery, hypertension treatment (BP ≥ 135/85 mmHg), and dyslipidemia treatment. Women with BMI ≥ 30 kg/m^2^ should take folic acid (400 μg or 5 mg/day) starting 1–3 months before conception and continuing through the first trimester
During pregnancy	Counseling: Inform about optimal ranges of GWG based on pre-pregnancy BMI Monitoring (mothers): Frequent control of weight, BP, FBG, urine tests; closer nutritional status monitoring if bariatric surgery was done Monitoring (Fetus): Periodic assessment of Doppler velocimetry, fetal growth, and size via ultrasound Treatment: Manage overt diabetes and hypertension (BP ≥ 140/90 mmHg outpatient, BP ≥ 135/85 mmHg at home) Screening for GDM: OGTT at 16–18 weeks; if negative, repeat at 24–28 weeks. Early screening for preeclampsia: Evaluate additional major risk factors in women with BMI ≥ 30 kg/m^2^; consider acetylsalicylic acid (100–150 mg/day) and calcium (600 mg/day) by the 16th week; increase fetal growth assessment frequency Labor and Delivery: Multidisciplinary management for women with BMI ≥ 35 kg/m^2^
After delivery	Cesarean delivery: Prescribe prophylactic antibiotics at the time of surgery; adjust pharmacologic thromboprophylaxis based on body weight Women with GDM: FBP monitoring soon after delivery. Preeclampsia: Monitor within the first 2 h and continue medical treatment post-discharge
After pregnancy	Breastfeeding: Support initiation and maintenance Screening: Check for postpartum mental health issues (depression, anxiety) Counseling: Emphasize importance of post-pregnancy weight loss to reduce future risks. Monitoring: If GDM occurred, repeat OGTT within 6–12 weeks. For preeclampsia, periodic BP assessments and a medical evaluation after 3 months
Postpartum follow-up care	GDM: Diabetes screening (OGTT, FPG, or HbA1C) from 4 to 12 weeks postpartum and every 1–3 years based on risk factors. OGTT is preferred early postpartum Hypertensive disorders: Physical examination, BP measurements, screening for cardiovascular risk factors (serum lipids, blood glucose) 6–12 weeks after birth and annually thereafter Liver Assessment: For women with MASLD Cancer Screening: For obesity-related cancers Renal Function: Laboratory testing of eGFR, BUN, creatinine, and microalbuminuria at 3 months postpartum and every 3–5 years Obstructive Sleep Apnea: Assessment and treatment Mental Health: Screening for postpartum depression and anxiety

BMI: body mass index; BP: blood pressure; FBG: fasting blood glucose; GWG: gestational weight gain; GDM: gestational diabetes mellitus; OGTT: oral glucose tolerance test; MASLD: metabolically associated steatotic liver disease; eGFR: estimated glomerular filtration rate; BUN: blood urea nitrogen

## Strength and limits

The key strength of this position statement of SIO lies in its comprehensive analysis of GO and the associated maternal and fetal complications. This extensive examination provides detailed insights into the risks and underlying mechanisms of GO. The robustness of the data supports its findings. However, this study is limited by the significant heterogeneity of the included studies. Variations in diagnostic criteria, measurement methods, and population characteristics across studies may impact the comparability and generalizability of the results. Additionally, the retrospective design of many included studies poses challenges for establishing causality.

## What is already known on this subject?

Prior to this study, extensive research indicated that gestational GO and excessive GWG are significantly associated with adverse maternal and fetal outcomes, including GDM, hypertensive disorders, preeclampsia, and fetal macrosomia. These adverse outcomes have both short-term and long-term health implications for both mothers and their offspring.

## What this study adds?

This study provides a more nuanced understanding of the impacts of GO on maternal and fetal health, emphasizing the increased risks associated with higher classes of obesity. It elucidates the differential effects of various obesity classes, advocating for more precise and individualized GWG recommendations for women with different obesity levels to mitigate adverse outcomes. The findings highlight the critical need for personalized lifestyle interventions, including tailored dietary and physical activity programs, to improve pregnancy outcomes. These results suggest that more individualized prenatal care guidelines and targeted interventions are essential for enhancing the health of pregnant women with GO and their children, potentially informing future policy and clinical practice guidelines.

## Data Availability

No datasets were generated or analysed during the current study.
